# IMPACT OF THE APPENDICEAL POSITION ON THE DIAGNOSIS AND TREATMENT OF PEDIATRIC APPENDICITIS

**DOI:** 10.1590/1984-0462/;2019;37;2;00012

**Published:** 2019-03-18

**Authors:** Belén Aneiros Castro, Indalecio Can Novillo, Araceli García Vázquez, Pedro Yuste Garcia, Eduardo Ferrero Herrero, Andrés Gómez Fraile

**Affiliations:** a Hospital 12 de Octubre, Madrid, Spain.

**Keywords:** Appendicitis, Children, Location, Laparoscopy, Apendicite, Crianças, Localização, Laparoscopia

## Abstract

**Objective:**

To investigate how symptoms vary according to the appendiceal position in pediatric patients and to demonstrate that the laparoscopic approach is safe and effective in any appendiceal location by comparing each location to another.

**Methods:**

The medical records of 1,736 children aged 14 or younger who underwent laparoscopic appendectomy over a period of 14 years were analyzed retrospectively. Patients were divided according to the position of the appendiceal tip into four groups: anterior, pelvic, retrocecal and subhepatic. The Kruskal-Wallis and chi-square tests were used with the Bonferroni correction, with a significant p<0.05.

**Results:**

The appendiceal location was anterior in 1,366 cases, retrocecal in 248 cases, pelvic in 66 cases and subhepatic in 56 cases. There were no significant differences between the groups in terms of patient age and gender. Abdominal pain was the only symptom with statistically significant differences between the groups. The rate of perforated appendicitis was higher in the subhepatic and pelvic positions. Intraoperative complications and conversions were not statistically significant. Technical difficulties and operative time were higher in subhepatic position. The rate of postoperative complications was similar between the different locations, except for bowel obstruction, which was higher in pelvic appendicitis.

**Conclusions:**

The clinical symptoms of appendicitis hardly ever change with the position of the appendix. The laparoscopic approach is safe and effective, regardless the appendiceal location.

## RESUMO

**Objetivo:**

Investigar como os sintomas variam de acordo com a posição do apêndice em pacientes pediátricos e demonstrar que a laparoscopia é segura e eficaz em qualquer posição do apêndice, comparando-as.

**Métodos:**

Os prontuários de 1.736 pacientes pediátricos com idade ≤14 anos submetidos à apendicectomia laparoscópica em um período de 14 anos foram analisados retrospectivamente. Os pacientes foram divididos de acordo com a posição do apêndice: anterior, pélvica, retrocecal e sub-hepático. Os testes de Kruskal-Wallis e do qui-quadrado foram usados com a correção de Bonferroni, sendo significante p<0,05.

**Resultados:**

A posição do apêndice era anterior em 1.366 casos, retrocecal em 248 casos, pélvica em 66 casos e sub-hepática em 56 casos. Não houve diferenças significativas entre os grupos quanto às variáveis idade e sexo. A dor abdominal foi a única variável com diferenças estatisticamente significantes entre os grupos. A taxa de apêndice perfurado foi superior nas posições sub-hepática e pélvica. As complicações intraoperatórias e a taxa de conversão não foram estatisticamente significativas. As dificuldades técnicas e o tempo cirúrgico foram superiores em posição sub-hepática. A taxa de complicações pós-operatórias foi semelhante entre as diferentes posições, exceto a obstrução intestinal, que foi superior em posição pélvica.

**Conclusões:**

Os sintomas da apendicite dificilmente variam com a posição do apêndice. A laparoscopia é segura e eficaz, independentemente da posição do apêndice.

## INTRODUCTION

The appendix is a narrow wormlike structure originated from the posteromedial wall of the cecum, at the site of coalescence of the three taenia coli, about 2 cm below the ileocecal valve.^1^ Its length varies from short (2 cm) to long forms (20 cm).^2^ The appendix is the most variable abdominal organ in terms of position and organ relations. There is not a consensus in the literature in relation to the different appendiceal positions in the abdominal cavity, thus many classifications have been proposed.^2^ ^,^ ^3^

The main goal of these classifications is to analyze the relationship between appendiceal positions and clinical symptoms. The typical presentation of appendicitis includes periumbilical pain located on the right quadrant, accompanied by anorexia and nausea. Fever and abdominal tenderness during physical examination are usually present.^4^ Some authors suggest that appendicitis in an unusual location may be presented with atypical symptoms and signs and is likely to be misdiagnosed or undiagnosed, resulting in a higher incidence of perforation and complications.^5^ ^,^ ^6^

The aim of our study is to investigate if there are differences in the clinical symptoms of appendicitis according to the appendiceal position in pediatric patients and to demonstrate that laparoscopic approach is safe and effective in any appendiceal location by comparing each location to another.

## METHOD

The medical records of children aged 14 years old or younger who underwent laparoscopic appendectomy at our institution over a period of 14 years were analyzed retrospectively.

We identified 1,736 patients with a diagnosis of acute appendicitis between January 2000 and December 2013. Information in the following sections was recorded: demographic data, historical findings, physical examination, surgical reports and postoperative care and complications. Informed consent was obtained from all individual participants included in the study.

Patients were divided into groups according to the appendiceal position. Although the relationship of the appendiceal base to the cecum is constant, the tip of the appendix may occupy several positions in relation to the cecum. The appendiceal situation may also be subject to changes, impaired even by the posture. We have defined the location of the appendix based on the situation of the appendiceal tip in the abdominal cavity during the laparoscopic surgery. Four positions have been described: anterior (the tip of the appendix lies anterior to the cecum, in the greater pelvis), retrocecal (the tip of the appendix lies posterior to the cecum, in the right iliac fossa), pelvic (the tip of the appendix lies in the lesser pelvis) and subhepatic (the tip of the appendix lies posterior to the cecum and reaches the subhepatic area). No left-sided appendicitis was found.

Statistical analyses were checked by SAS 9.3 software (SAS Institute Inc., Cary, NC). The Kruskal-Wallis test was used to compare continuous variables and the chi-square test was performed on patients’ categorical data. The Bonferroni correction for multiple comparisons was applied. A probability of a p<0.05 was accepted as indicating statistical significance. The study was approved by the ethics committee of the institution.

## RESULTS

Among the overall patients (n=1,736), the appendiceal location was anterior in 1,366 cases (78.7%), retrocecal in 248 cases (14.2%), pelvic in 66 cases (3.8%) and subhepatic in 56 cases (3.3%). The mean age of patients was 8.9±3.2 years. There were no significant differences between the groups in terms of patient age (p=0.573) and gender (p=0.238). The demographic data are summarized in Table 1.

**Table 1:** Demographic data.

	Anterior (n=1,366)	Pelvic (n=66)	Retrocecal (n=248)	Subhepatic (n=56)	p-value
Age (years)	8.9±3.2	8.9±3.3	9.1±3.1	8.6±3.0	0.573
Sex					
Male	871 (63.8%)	39 (59.1%)	142 (57.3%)	36 (64.3%)	0.238
Female	495 (36.2%)	27 (40.9%)	106 (42.7%)	20 (35.7%)	

The comparison of clinical symptoms and signs between the different groups are shown in Table 2. The mean duration of symptoms and vomiting were not statistically significant. In addition, diarrhea, urinary symptoms and fever became not statistically significant when a Bonferroni correction for multiple comparisons was applied. Findings of abdominal pain were significantly associated with the position of the appendix. There was a significant difference in the location of the pain between the pelvic and the retrocecal position after Bonferroni correction (p=0.024).

**Table 2:** Comparison between clinical symptoms and signs.

	Anterior (n=1,366)	Pelvic (n=66)	Retrocecal (n=248)	Subhepatic (n=56)	p-value
Mean duration of symptoms (days)	1.3	1.5	1.1	1.1	0.396
Vomiting	66.6%	69.7%	68.7%	75.9%	0.474
Diarrhea	14.4%	18.1%	8.9%	7.4%	0.040
Urinary symptoms	8.4%	4.5%	4%	0	0.010
Temperature					
Afebrile	37.2%	25.7%	42.5%	25.9%	0.040
Mild fever	24.2%	27.3%	26.3%	24.1%	
Fever (≥38ºC)	38.6%	47%	31.2%	50%	
Abdominal pain					
Right iliac fossa	81.8%	71.4%	88.8%	81.4%	0.022*
Widespread	14.1%	22.2%	7.4%	14.8%	
Other locations	4.1%	6.4%	3.8%	3.8%	

The rate of perforation in the present study was 11.4%. Pelvic and subhepatic groups were more likely to have a perforated appendix (18.1 and 16%, respectively) than anterior and retrocecal groups (11.7 and 7.2%, respectively). A phlegmonous appendix was found in 57% of cases in the anterior group, 43.9% of cases in the pelvic group, 59.6% of cases in the retrocecal group and 35.7% of cases in the subhepatic one, whereas a gangrenous appendix was found in 24.9% of cases in the anterior group, 33.3% of cases in the pelvic group, 26.2% of cases in the retrocecal group and 44.6% of cases in the subhepatic one. After Bonferroni correction, there were significant differences in the aspect of the appendix between anterior and subhepatic groups (p=0.022), and retrocecal and subhepatic groups (p=0.025).

Surgical records are summarized in Table 3. The rates of intraoperative complications (rupture of appendix and appendiceal bleeding) and conversion were not statistically significant. There were significant differences in the technical difficulties between anterior (1.3%), pelvic (0%) and subhepatic groups (7.1%), and in the operative time between anterior and retrocecal groups (p<0.0001), anterior and subhepatic groups (p<0.0001), pelvic and subhepatic groups (p=0.0006), and retrocecal and subhepatic groups (p=0.009)

**Table 3:** Surgical records.

	Anterior (n=1,366)	Pelvic (n=66)	Retrocecal (n=248)	Subhepatic (n=56)	p-value
Rupture of appendix	8%	9%	11.6%	14.2%	0.130
Appendiceal bleeding	2.4%	3%	2.8%	1.7%	0.952
Technical difficulties	1.3%	0	3.6%	7.1%	0.0008*
Conversion	1%	0	0.8%	1.7%	0.768
Mean operative time (minutes)	55.6	56.8	63.2	75.6	<0.0001*

*Statistically significant after Bonferroni correction.

Postoperative outcomes are shown in Table 4. The antibiotic therapy was different after Bonferroni correction between retrocecal (3.2 days) and subhepatic (4.4 days) groups (p=0.006), as well as the oral re-feeding between pelvic (1.9 days) and retrocecal (1.3 days) groups (p=0.02). Moreover, there were statistical differences in the hospital stay between anterior (4.9 days) and subhepatic (6.1 days) groups (p=0.006) and retrocecal (4.5 days) and subhepatic groups (p=0.02). The rates of intra-abdominal abscess and wound infection were not statistically significant. However, the rate of bowel obstruction was different between anterior (1.4%) and pelvic (9%) groups (p<0.0001) and pelvic and retrocecal (0%) groups (p<0.0001).

**Table 4:** Postoperative outcomes.

	Anterior (n=1,366)	Pelvic (n=66)	Retrocecal (n=248)	Subhepatic (n=56)	p-value
Antibiotic therapy (days)	3.4	4.4	3.2	4.4	0.001*
Oral re-feeding (days)	1.5	1.9	1.3	1.5	0.007*
Analgesic therapy (days)	2.6	3.3	2.8	2.8	0.05
Hospital stay (days)	4.9	6.5	4.5	6.1	0.009*
Abdominal abscess	6.8%	10.6%	5.6%	14.2%	0.088
Wound infection	1.9%	0	0.8%	5.3%	0.083
Bowel obstruction	1.4%	9%	0	0	<0.0001*

*Statistically significant after Bonferroni correction.

## DISCUSSION

Atypical locations of the appendix have been reported to be anywhere in the abdominal cavity, especially in cases of intestinal malrotation.^1^ There is a controversy among the authors regarding the different positions of the appendix and, thus, there are a lot of classifications in the literature.^2^ ^,^ ^3^ ^,^ ^4^ However, most of these studies have been performed at autopsies or open surgeries in adults. We have developed a classification based on the situation of the appendiceal tip during laparoscopic examination.

Acute appendicitis is still a difficult diagnosis. Several series have reported that an abnormal location of the appendix is likely to have an atypical clinical presentation, resulting in a higher incidence of misdiagnoses and complications.^5^ ^,^ ^6^ Other authors, however, reported that the appendiceal position does not alter the presentation of the appendicitis.^7^ ^,^ ^8^ In our study, there were no statistically significant differences in the clinical symptoms and signs between the groups, except for the location of the abdominal pain. Appendicitis may mimic other acute abdominal diseases, so it should be considered in the differential diagnosis of diseases like mesenteric adenitis, urinary tract infection, Meckel’s diverticulum, cholecystitis or gynecological pathology in females.^4^ ^,^ ^6^

The rate of perforated appendicitis is higher in children than in adults and varies from 5 to 75%.^9^ ^,^ ^10^ Different Clinical Decision Rules (CDR), like the Pediatric Appendicitis Score (PAS) and the Alvarado score, have been developed in order to improve the accuracy of appendicitis diagnosis in children and to prevent perforation of the appendix. Despite this, diagnosis of appendicitis in pediatric patients is still a clinical challenge because of atypical presentations in this population.^10^ ^,^ ^11^ Different risk factors associated with perforation have been reported, including younger age and longer duration of complaints.^10^ In our series, pelvic and subhepatic groups had a higher rate of perforated appendix (18.1 and 16%, respectively) than anterior and pelvic groups. However, the mean duration of the symptoms and the mean age of the patients were similar in the four groups.

The laparoscopic approach is commonly used to perform appendectomies in pediatric patients. This approach allows surgeons to inspect the abdominal cavity and to exclude alternative diagnoses, especially if the appendix is normal.^12^ ^,^ ^13^ In addition, laparoscopic appendectomy in rare anatomical positions is a better option than the open technique because, once the camera is introduced and the appendix is located, the surgeon chooses the trocars and decides where to put them.^14^ ^,^ ^15^ In our study, the mean operative time was statistically longer in retrocecal and subhepatic groups. There were technical difficulties in 7.1% of subhepatic cases. This could happen because there were more peritoneal adhesions in posterior locations and the appendiceal dissection was more difficult. However, overall intraoperative complication rates showed no statistically differences between the groups.

We have also found that antibiotic therapy, oral re-feeding and hospital stay were higher in subhepatic and pelvic groups. This was not surprising because, as mentioned before, there were more complicated appendicitis in these groups. The rates of intra-abdominal abscess and wound infection were similar among groups, while the rate of bowel obstruction was higher in the pelvic group. The vast majority of bowel obstruction is due to intraperitoneal adhesions developed as a response to peritoneal trauma. Laparoscopic approach has demonstrated to reduce the presence of adhesions compared with open surgeries.^16^ ^,^ ^17^ In our series, the pelvic group needed no conversion, so we assumed that there was more manipulation of the bowel to expose the appendix in this group.

The main limitation of this study is that it was based on a retrospective analysis and it was conducted in a single institution. In addition, the position of the appendix was described according to the perception of the surgeon during the surgery and, in some cases, this may be quite subjective.

In conclusion, the clinical symptoms of appendicitis hardly change with the position of the appendix. The rate of postoperative complications was similar between the different locations, except the bowel obstruction, which was more frequent in pelvic appendicitis. The laparoscopic approach is safe and effective regardless the location of the appendix and it allows surgeons to guide trocar placement according to appendiceal position, improving the visualization and the exposure of the appendix and avoiding unnecessary incisions.
