# ERRATUM

**DOI:** 10.1590/1984-0462/;2019;37;2;00012erratum

**Published:** 2020-01-17

In the manuscript “Impact of the appendiceal position on the diagnosis and treatment of pediatric appendicitis”, DOI: 10.1590/1984-0462/;2019;37;2;00012, published in the Rev Paul Pediatr. In press 2019. Epub Mar 18, 2019:

Where it reads:

Indalecio Can Novillo

It should read:

Indalecio Cano Novillo

